# Force Generation in the Coiling Tendrils of *Passiflora caerulea*

**DOI:** 10.1002/advs.202301496

**Published:** 2023-08-06

**Authors:** Frederike Klimm, Thomas Speck, Marc Thielen

**Affiliations:** ^1^ Plant Biomechanics Group @ Botanic Garden University of Freiburg Schänzlestraße 1 D‐79104 Freiburg Germany; ^2^ Freiburg Center for Interactive Materials and Bioinspired Technologies (FIT) Georges‐Köhler‐Allee 105 D‐79110 Freiburg Germany; ^3^ Freiburg Materials Research Center (FMF) Stefan‐Meier‐Straße 21 D‐79104 Freiburg Germany; ^4^ Cluster of Excellence livMatS @ FIT – Freiburg Center for Interactive Materials and Bioinspired Technologies University of Freiburg D‐79110 Freiburg Germany

**Keywords:** climbing plants, coiling, Passiflora, plant biomechanics, tendrils

## Abstract

Tendrils of climbing plants coil along their length, thus forming a striking helical spring and generating tensional forces. It is found that, for tendrils of the passion flower *Passiflora caerulea*, the generated force lies in the range of 6–140 mN, which is sufficient to lash the plant tightly to its substrate. Further, it is revealed that the generated force strongly correlates with the water status of the plant. Based on a combination of in situ force measurements with anatomical investigations and dehydration‐rehydration experiments on both entire tendril segments and isolated lignified tissues, a two‐phasic mechanism for spring formation is proposed. First, during the free coiling phase, the center of the tendril begins to lignify unilaterally. At this stage, both the generated tension and the stability of the form of the spring still depend on turgor pressure. The unilateral contraction of a bilayer as being the possible driving force for the tendril coiling motion is discussed. Second, in a stabilization phase, the entire center of the coiled tendril lignifies, stiffening the spring and securing its function irrespective of its hydration status.

## Graphical abstract

Climbing plant tendrils coil into a spring‐like structure and thereby pull the plant closer to its support. Here, the forces generated by this coiling are reported, which correlate with the plant's water status. Based on mechanical, anatomical, and morphological data, a two‐phasic process for the spring formation is proposed, which consists of an active coiling phase and a stabilization phase.

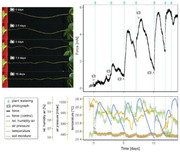

## 1 Introduction

**Figure 1:**
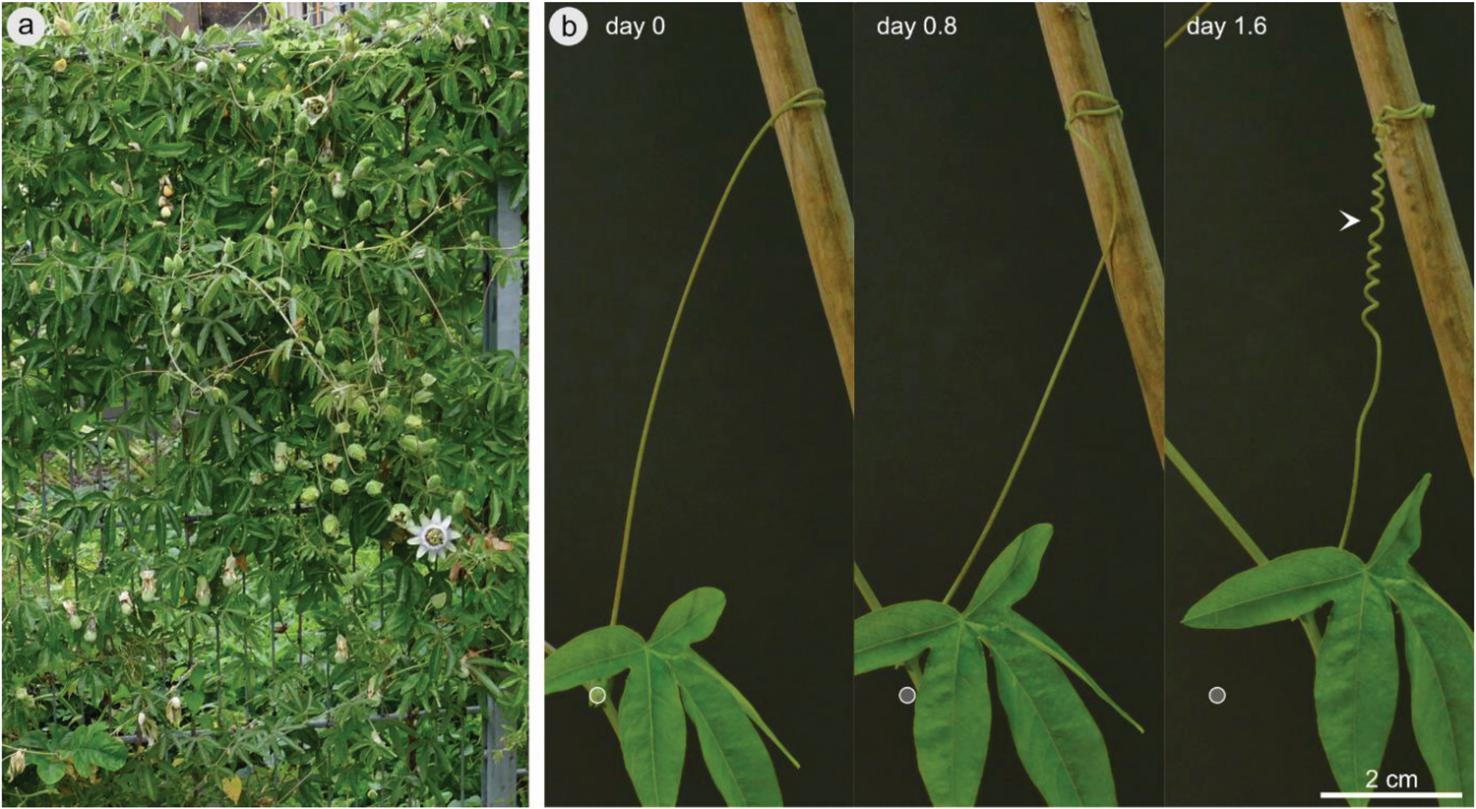
Climbing passion flower *Passiflora caerulea*. Habitus of *P. caerulea* climbing on a metal fence in the outdoor area of the Botanic Garden of the University of Freiburg a). Images of a free‐coiling *P. caerulea* tendril lashing the plant stem closer to its support (white circle indicates reference position of the node at day 0) and forming a spring‐like shape consisting of two helices of opposite handedness connected by an inversion point (perversion, arrowhead) b). See also time‐lapse Video S1 (Supporting Information).

Plants as rooted sessile organisms are not usually associated with movement. They do however move substantially, as is particularly evident in climbing plants (e.g.,^[^ ^1^ ^]^). Instead of producing a stiff self‐supporting trunk, these plants rely on supporting host structures to reach upward to the light (e.g.,^[^ ^2^, ^3^ ^]^) (**Figure** **1**a). They attach to their supports, for instance, by tendrils. These filamentary organs are sensitive to contact^[^ ^1^ ^]^ and either possess adhesive discs at their tips, as in, for example, the tendrils of *Parthenocissus tricuspidata* ^[^ ^4^ ^]^ or *Passiflora discophora*,^[^ ^5^ ^]^ or “grasp” slender supports by coiling around them (a process termed as contact coiling, e.g.,^[^ ^6^ ^]^). Eventually, the tendrils coil along their length axis in a movement referred to as free coiling (e.g.,^[^ ^6^ ^]^). They do so by developing a length difference between their prospective convex and concave sides (e.g.,^[^ ^7^ ^]^), thereby creating an intrinsic curvature and eventually resulting in helical coiling. Since the tendril's ends are fixed in position, free coiling results in a particular spring‐like shape with a minimum of one perversion, which links two helices of opposite handedness (Figure 1b).^[^ ^8^, ^9^ ^]^

Several mechanisms have been suggested to cause the length difference leading to tendril curvature and eventually to free or contact coiling, including differential growth, turgor changes,^[^ ^1^, ^6^, ^10^, ^11^ ^]^ and the contraction of G‐fibers.^[^ ^12^ ^]^ G‐fibers are fiber cells that, in addition to having the typical primary and secondary cell wall layers, possess a gelatinous‐ or G‐layer that enables them to contract (e.g.,^[^ ^13^, ^14^ ^]^). A prominent instance of the occurrence of these fibers is the tension wood in trees (e.g.,^[^ ^14^, ^15^ ^]^) and contractile roots.^[^ ^16^ ^]^ As mentioned above, this type of fiber is also associated with twining and coiling in many climbing plant species.^[^ ^12^, ^13^, ^17^, ^18^, ^19^ ^]^ However, the correct identification of the G‐layer in some of the cited works has been brought into question.^[^ ^13^ ^]^ In tendrils, the presence of contractile G‐fibers suggests a straightforward mechanism for bending and thus coiling, notably by means of a bilayer that combines one contractile layer with a non‐ or, at least, a less contractile antagonistic layer. Further, the contractile force of G‐fibers is suggested to vary because of differential lignification, resulting in variations in hydrophobicity and, thus, in water repulsion.^[^ ^18^ ^]^ This suggestion is consistent with the observation that the tendrils of various Cucurbitaceae species and the extracted lignified tissue ribbons therefrom coil further upon drying.^[^ ^18^, ^20^ ^]^

The described free coiling movement concomitantly causes an axial shortening of the tendril. The tensional force generated in this process reduces the distance between the tendril's insertion point at the climber's stem and its anchorage point to the support, thus pulling and lashing the whole plant closely to its support structure (e.g.,^[^ ^7^ ^]^) (Figure 1b). The force generated by the free coiling movement, denoted as the coiling force in the following, was measured for tendrils of *Passiflora* and *Cucurbita* species many years ago by MacDougal;^[^ ^7^, ^10^ ^]^ he used various dynamometer setups and found that the coiling force exceeded the plant stem's weight and that it decreased in a wilted plant. More recently, Eberle et al.^[^ ^21^ ^]^ employed a pendulum setup to measure the coiling force generated by *Luffa cylindrica*. Neither of these setups, however, included the continuous measurement of the force development. Continuous force‐time curves for the coiling force generated by *Luffa cylindrica* have been reported by Liou et al.,^[^ ^22^ ^]^ but their cantilever wire setup did not discriminate the coiling force from the force exerted by the plant weight.

In this study, we present a custom‐built setup that allows, for the first time, in situ measurements of tendril coiling forces over time, isolated from plant weight and in exact axial alignment. We have studied tendrils of *Passiflora caerulea* as a representative of the passion flower genus. Like the vast majority of the more than 500 passion flower species, *P. caerulea* is a vigorous climber that uses axillary unbranched tendrils for attachment via contact coiling.^[^ ^23^, ^24^ ^]^ It is native to Argentina, Brazil, and Paraguay.^[^ ^23^ ^]^ To complement the coiling force measurements and to gain a deeper understanding of the mechanism involved in force generation, we have analyzed tendril anatomy at various stages in ontogeny. Inspired by MacDougal's^[^ ^10^ ^]^ observation that the coiling force decreased in wilting plants and by Gerbode et al.’s^[^ ^18^ ^]^ findings that both the entire cucumber tendril and the isolated fiber ribbons therefrom continue coiling even under drying, we have analyzed the dependence of the coiling force on the hydration status of *Passiflora caerulea* plants. We have therefore complemented our in situ measurements with dehydration‐rehydration experiments on both entire tendrils and isolated lignified tissues.

In summary, this study aims to answer the following questions:

1. Do *P. caerulea* tendrils develop sufficient force to support and move the plant stem?
2. Is this force sensitive to fluctuations in water availability?
3. How do the different tissues contribute to the formation of the tendril spring?

## 2 Results

### 2.1 Coiling Force Measurements

**Figure 2:**
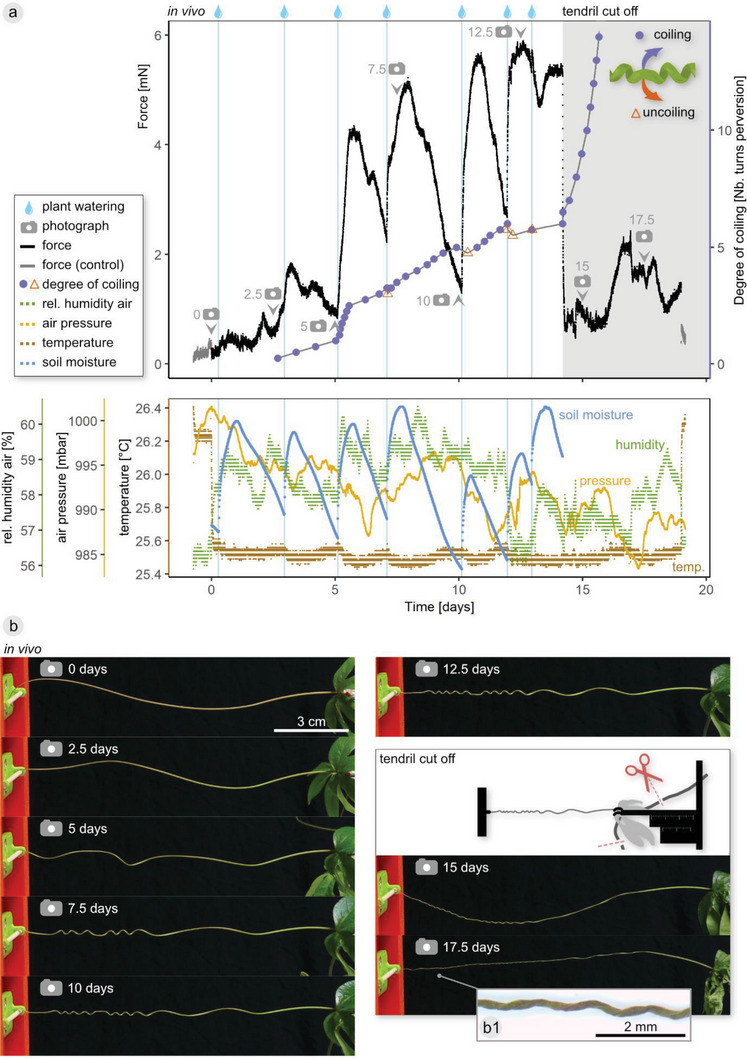
Tensile force generated during free coiling of a *P. caerulea* tendril. Force and environmental conditions and the degree of coiling or uncoiling are plotted over time a). Drop icons indicate the watering of the plant pot. After 14.2 days, the plant stem was cut apically and basally from the tendril insertion point, leaving the tendril fixed in an unaltered position in the experimental setup during drying. Photograph icons indicate video stills in b), inset b1) shows magnified view of dried tendril at day 17.5. See Video S2 (Supporting Information) for a synchronized video of force data and time‐lapse video. Control indicates force before tendril was put in setup and after it was removed.

The coiling force of four *P. caerulea* tendrils was measured. From these, three measurements served primarily for studies of the maximum force produced and one measurement additionally served for investigating the influence of the plant's hydration status on the coiling force. For this measurement, soil humidity was tracked continuously during tendril coiling, the coiled tendril was cut off from the plant and left to dry within the measurement setup and the coiling motion during the measurement was quantified (**Figure** **2**).

The coiling force in the four tendrils reached maximum total values of 6, 11, 49, and 140 mN, respectively. The force initially remained low but increased when the tendrils started to coil (Figure 2; and Figure S1, Supporting Information). Whereas in the two tendrils generating the highest maximum forces (Figure S1a,b, Supporting Information), the force remained high with only relatively small “jumps” upward followed by a slower de‐ or increasing force, the force generated by the other two tendrils was subject to stronger fluctuations (Figure 2; and Figure S1c, Supporting Information). In general, fluctuations in temperature, relative humidity, or air pressure in the phytochamber were small and did not reflect in the force‐time curves.

The first sharp increase in force was correlated with an increased coiling movement expressed by a faster rotation of the perversion (Figure 2). Over time, the entire tendril axis coiled and formed one or more perversions, except for a basal segment that remained straight. Increases in force that occurred once the tendril had slightly coiled, i.e., established a connection under tension to the sensor, correlated well with the watering of the plant (Figure 2; and Figure S1 (Supporting Information) for the other three measured tendrils). An increase in force was typically noticeable within less than 10 min after watering.

Once the tendril was coiled helically, both the increase and decrease in force was clearly correlated with the variation in soil moisture (Figure 2). Moreover, variations in the coiling motion coincided with watering: at the third watering point (day 5.1), the coiling speed increased sharply, whereas at the subsequent watering points (day 7.1, 10.1, 12.0, 12.9), the perversion slightly uncoiled (less than half a turn). At watering point five (day 10.1), the tendril had visibly drooped, and in parallel to the uncoiling of the perversion, it tightened again, i.e., the drooping tendril axis straightened. When the plant stem was cut apically and basally from the tendril insertion point at day 14.2, leaving the tendril fixed in an unaltered position in the experimental setup to dry off, the force started sharply to decrease within minutes. The coiled part of the tendril drooped, the coil shrank in diameter, and the number of coils increased quickly, causing the tendril axis to be lifted back upward.

When a segment of the dehydrated tendril was submerged in tap water, it rehydrated and had an appearance similar to that before the tendril was cut from the plant stem (Figure S2, Supporting Information).

### 2.2 Tendril Anatomy

**Figure 3:**
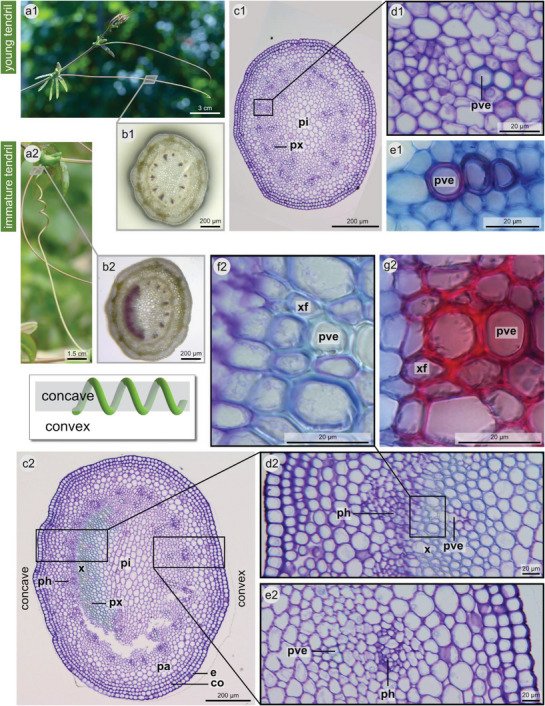
Tendril anatomy in the young (uncoiled) state and the immature (not yet fully coiled) state. Cross‐sections through a young tendril a1) show evidence for lignification only in the primary vessel elements b1–e1). In the coiling but still immature tendril a2), secondary xylem with lignified cells is present at the concave side but not at the convex side b2–g2). b1,b2) Free‐hand sections, stained with phloroglucinol‐hydrochloric acid (red color indicates lignification). c1–d1,c2–f2) Thin sections of embedded plant material stained with toluidine blue (blue color indicates lignification). e1,g2) Cryotome sections stained with astrablue‐safranin (red color indicates lignification, blue color indicates presence of cellulose). e = epidermis, co = collenchyma, pa = parenchyma, ph = phloem, pi = pith, pve = primary vessel element, px = primary xylem, x = secondary xylem, xf = xylem fibers. For reference sections that were used to determine the orientation (convex/concave side), see Figure S3 (Supporting Information).

In the tendril cross‐section, primary vascular bundles are arranged elliptically around the pith. The vascular cylinder is surrounded by the cortex, which comprises the parenchyma and collenchyma, and the epidermis. Within the vascular bundles, primary vessel elements have lignified thickened cell walls that stain red after treatment with phloroglucinol‐hydrochloric acid (P‐HCl) (**Figure** **3**b1,b2), light blue after treatment with toluidine blue (TB) (Figure 3c1–d1 and c2–f2), and red after treatment with astrablue‐safranin (A‐S) (Figure 3e1,g2).

**Figure 4:**
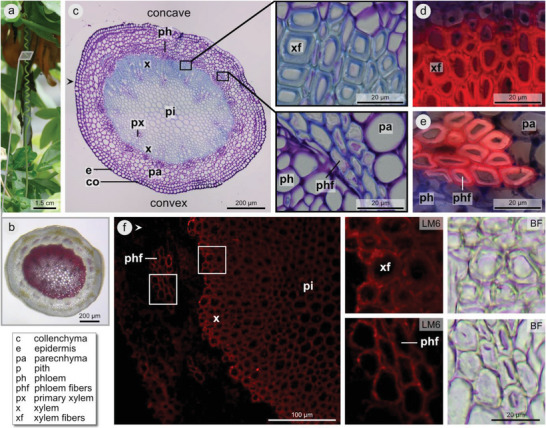
Tendril anatomy in the mature (fully coiled) state. Cross‐sections of a mature tendril a) show that the lignified secondary xylem is more pronounced on the concave tendril side b,c). Astrablue‐safranin staining indicates that all cell wall layers are lignified in xylem fibers d). Thick‐walled phloem fibers (phf) are present at the concave tendril side, with lignified outer cell wall layers e). The red fluorescent signal (excitation: 510–550 nm) in f) indicates localization of LM6 antibody, with additional bright‐field view (BF) at high magnification showing the cell wall thickness of xylem and phloem fibers for comparison. b) Free‐hand section, stained with phloroglucinol‐hydrochloric acid (red color indicates lignification). c) Thin section of embedded material stained with toluidine blue (blue color indicates lignification), with magnified insets. d,e) Cryotome sections stained with astrablue‐safranin (red color indicates lignification, blue color indicates cellulose). f) Sections immunolabeled with antibody LM6, arrowhead indicates orientation of section compared with the section presented in c). For reference sections that were used to determine the orientation (convex/concave side), and for a control section without antibody, see Figure S3 (Supporting Information).

Based on the anatomical organization and differentiation, we distinguish three ontogenetic stages: young tendrils (uncoiled, Figure 3a1–e1), immature tendrils (not yet fully coiled, Figure 3a2–g2), and mature tendrils (fully coiled, **Figure** **4**). In young tendrils, the vessel elements of the primary xylem are the only cells with (slightly) lignified cell walls (Figure 3c1–e1), and no secondary xylem is present.

**Figure 5:**
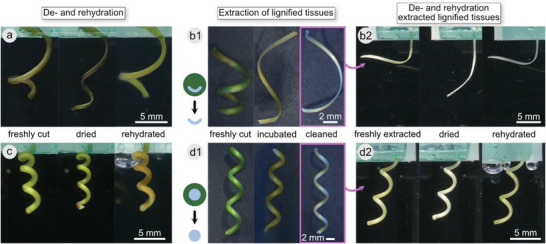
Tendril shape variations under de‐ and rehydration and extraction of lignified tissues. The effect of drying and rehydration on the shape of a coiled tendril segment is demonstrated for an immature tendril a) and a mature tendril c) and for extracted lignified tissues from an immature b) and a mature tendril d). Stereomicroscopic pictures in the center b1,d1) show a freshly cut tendril segment that was then incubated with the enzyme Driselase to digest parenchymatic tissues and cleaned to remove loose tissue remains. The clean sample was then dried and rehydrated b2,d2). Images show the extracted samples b2,d2) or freshly cut samples a,c) in air, dried for 2 days in air, and rehydrated for 2 days submerged in water. See also time‐lapse videos of dehydration and rehydration in Video S3 (Supporting Information).

In contrast, immature tendrils are characterized by thick‐walled lignified xylem fibers (Figure 3b2–g2). These fibers lie at the periphery of the primary vessel elements on the concave inner side of the tendril. On this side, the cells that are located in‐between the primary vessel elements also have thickened lignified cell walls. In addition, the most peripheral pith cells located on the concave side are lignified and thicker‐walled than are those in the center of the tendril (Figure 3d2), whereas the remaining pith parenchyma cells do not stain for lignin and are in general thin‐walled (Figure 3c2,e2). Thick‐walled phloem fibers are not present in the cross‐section. When treated with the cell‐wall‐degrading enzyme Driselase to remove tissues without lignified cell walls, a tissue with a crescent shaped cross‐section is obtained representing a fiber ribbon (**Figure** **5**b1).

Mature tendrils are characterized by a central cylinder comprising lignified xylem and pith (Figure 4b,c), and this central cylinder can be isolated by treatment with Driselase (Figure 5d1). The term “central cylinder” is used here as a descriptive term, rather than in the strict botanical sense. The secondary xylem has more layers of thick‐walled xylem fibers on the concave side than on the convex side of the tendril coil (Figure 4c,d). On the concave side, thick‐walled phloem fibers are visible in which the outer cell wall layer stains blue (TB) (Figure 4c) or red (A‐S) (Figure 4e), whereas the thick inner layer remains unstained (TB) (Figure 4c) or stains only slightly red (A‐S) (Figure 4e) indicating that only the outer cell wall layer is lignified. On the convex side, we have observed that thick‐walled lignified phloem fibers are absent (Figure 4c), or that the fibers are lignified but lack the thick inner cell wall layer devoid of lignin (Figure 4b; and Figure S3, Supporting Information). Antibody LM6, which targets (1,5)‐α‐L‐arabinosyl residues found in the arabinan components of certain pectic polymers such as rhamnogalacturonan‐I (Megazyme Ltd, Bray, Ireland), binds within both xylem and phloem fibers and within the pith. The thickened inner cell wall layer does not stain within phloem fibers (Figure 4f, comparison fluorescent and bright field view).

In the immature state, a large part of the tendril tissues collapsed when the tendril dehydrated. In comparison with the cross‐section of a fresh (fully turgescent) immature tendril (Figure 3c2), the cross‐section of a tendril that had been left to dry out in the coiling force setup (Figure 2b) is distorted, and the exact tissue arrangement is hard to detect (Figure S4, Supporting Information). Only a small part of the cross‐section stains light blue (TB), and the cross‐section has a central cavity (Figure S4c–e, Supporting Information). In the basal straight part of the tendril, only the epidermis, collenchyma, cortex, and phloem had however collapsed, and the cross‐section exhibits an intact central cylinder formed by a ring of mostly light‐blue‐stained (TB) and (in the outer part of the ring) thick‐walled cells around a central cavity (Figure S4f, Supporting Information).

### 2.3 De‐ and Rehydration Behavior

When an immature tendril segment was left to dry out, it underwent a complex shape‐changing pattern and eventually coiled slowly, while its cross‐section flattened (Figure 5a; and Video S3, Supporting Information). Rehydrated, the tendril resumed a shape close to that of the initial one. When the lignified fiber ribbon from such an immature tendril was isolated by Driselase treatment, its coiled shape loosened, and only a smaller degree of curvature was maintained (Figure 5b1). Left to dry, the isolated ribbon bent open. When rehydrated, it resumed its initial slightly curved shape (Figure 5b2).

When a mature tendril was left to dry, it shrank in diameter and coiled by an additional small extent (Figure 5c; and Video S3, Supporting Information). Upon rehydration, it resumed its initial shape. When the lignified central cylinder from such a tendril was isolated by Driselase treatment, it remained unaltered in its coiled shape (Figure 5d1). Upon drying, the isolated cylinder contracted and coiled by an additional small extent. When rehydrated, it resumed its initial shape (Figure 5d2).

### 2.4 Fresh Weight

The plant stems with leaves had a mass of 0.7 ± 0.2 g per node (mean ± standard deviation), i.e., per tendril, equivalent to 6.9 mN. The flower's mean mass was 3.2 ± 0.5 g, equivalent to 31.4 mN.

## 3 Discussion

We found that on coiling, the tendrils of *P. caerulea* generate tensional forces ranging from 6 to 140 mN. In nature, the tension caused by coiling transcribes to a shortening of the tendril by as much as one‐third of its straight length for *P. caerulea*.^[^ ^7^ ^]^ This is in good agreement with measurements performed by MacDougal,^[^ ^7^, ^10^ ^]^ who used a so‐called Vöchting's dynamometer and measured tensional forces between 30 and 100 mN for tendrils of *P. caerulea* and *P. pfordti*. Liou et al.,^[^ ^22^ ^]^ using a setup in which the shortening tendril bent a wire from which force was then extrapolated, measured tensile forces of 170 mN for *Luffa cylindrica* tendrils, whereas Eberle et al.^[^ ^21^ ^]^ measured only 3.4 ± 0.2 mN of tensional force for the same species, by using a pendulum apparatus. The latter eliminated the influence of the plant's own weight on the measurements by restraining the plant stem. Our study is the first that allows continuous measurement of the tensional force that coiling tendrils are able to generate when both ends are fixed. As the plant stem produces about 7 ± 2 mN of weight force per node and thus per tendril, the generated tensional force indeed enables the tendrils to move the plant stem against gravity and thus to lash the plant to its support.

Tendrils coil because of their intrinsic curvature (e.g.,^[^ ^8^ ^]^), which is generated by a length difference between the sides of the tendril, with the convex side being longer than the concave side. Since contractile G‐fibers are common in climbing plant tendrils,^[^ ^12^, ^17^ ^]^ it is safe to assume that contraction is involved in creating the length difference. The arrangement of G‐fibers in tendrils varies and includes both centri‐symmetric arrangements (e.g., a hollow cylinder in tendrils of *Smilax rotundifolia*)^[^ ^12^ ^]^ and bilateral symmetric arrangements (e.g., a G‐fiber ribbon on the concave side of cucurbit tendrils).^[^ ^12^, ^18^ ^]^ Whereas contraction of a centri‐symmetric tissue will not lead to curvature and coiling, the presence of a bilateral symmetric tissue organization suggests a straightforward explanation for tendril coiling, namely coiling via a bilayer composed of an actuating contractile layer and a second antagonizing non‐ (or less) contractile resistance layer. The coiling and formation of perversions by such a bilayer can be visualized, for example, by stretching silicone rubber, adding a second layer of relaxed silicone, and releasing the thus fabricated bilayer strips (e.g.,^[^ ^18^ ^]^).

In *P. caerulea* tendrils, xylem fibers develop unilaterally at the concave side of the tendril during coiling, and a bilateral tissue organization thus appears. This concurs with observations by MacDougal^[^ ^25^ ^]^ who described, more than a century ago, the unilateral development of wood at this same side of *P. caerulea* tendrils. The determination of this unilateral development might be triggered, for example, by a contact or gravitational stimulus or might be anatomically predetermined. The observation that the thickened cell walls of these xylem fibers are immunolabeled with the LM6 antibody targeting i.a. RG‐I, a pectic polysaccharide found in plant primary cell walls and in the G‐layer of fiber cells,^[^ ^17^, ^26^, ^27^ ^]^ shows that these fibers could indeed be G‐fibers. The labeling includes the thick‐walled cells in between the vascular bundles, which are unlikely to be fibers but most probably provide mechanical support. However, the cell walls of the xylem fibers are fully lignified, which is untypical for G‐fibers.^[^ ^13^ ^]^ Nevertheless, the degree of lignin cross‐linking might still be low in the potential G‐layer, allowing for G‐fiber contraction and movement of the plant organ. If the xylem fibers are G‐fibers, then the dehydration‐rehydration experiments suggest that the xylem fiber ribbon represents only one individual layer (the actuating layer) of the above‐discussed bilayer necessary for tendril coiling. An extracted fiber ribbon from a *P. caerulea* tendril does not coil or bend when dried, which means that the bilayer is not “complete.” A possible driving motor for the coiling of *P. caerulea* tendrils is thus the existence of a bilayer composed of an eccentrically positioned xylary G‐fiber ribbon as an actuating contractile layer with the compression‐resistant parenchyma acting as a resistance layer. The thick parenchymatous tissue layer composed of cortex and pith on the convex side of the fiber ribbon resists contraction more strongly than the thinner layer on the concave side (cortex only), and the tendril thus bends and coils. This is unlike the situation in cucumber tendrils where the G‐fiber ribbon alone coils as it dries, forming an asymmetrically contracting bilayer that imposes its coiled shape on the surrounding soft tissue.^[^ ^18^ ^]^

Even though mature tendrils show an overall more centri‐symmetrical tissue arrangement, thick‐walled phloem fibers only develop on the concave tendril side. The observation of a thickened innermost cell wall layer devoid of lignin in these fibers supports the hypothesis that they are extraxylary G‐fibers, as has been previously suggested for tendrils and stems of *Passiflora*.^[^ ^18^, ^19^ ^]^ However, the development of this potential G‐layer occurs too late to contribute to the main coiling process, as we have not observed it in the immature state during coiling but only in the mature, fully coiled state. Rather, the phloem fibers might support the coiled structure and might be formed as a reaction to the increasing tensional load on the tendrils as the plant grows and increases in weight.

To confirm that the xylem and/or phloem fibers are indeed G‐fibers, more thorough and specific histological investigations are needed, which are far beyond the scope of the present study, but the subject of ongoing research. These should include more comprehensive immunolabeling experiments (cf., for example,^[^ ^12^, ^13^ ^]^), since the LM6 antibody is not specific enough for G‐layer detection, and investigations into the cross‐linking degree of lignin in the fiber cell walls. Additionally, microfibril angles in the cell wall should be measured, since the microfibril angle in G‐fiber cells varies characteristically between the cell wall layers (e.g.,^[^ ^16^ ^]^).

The tension generated by tendril coiling correlates strongly with the watering of the plant, which we assume is translated (with a certain delay) into turgor pressure within the cells of the tendril. During coiling, the tendrils remain in an immature state and consist mostly of thin‐walled parenchyma cells. Parenchymatous tissues mechanically act as hydrostats, whose cell walls are placed under tension by the pressure generated within the walls.^[^ ^28^ ^]^ The thus “inflated,” fully turgescent, parenchyma cells are compression‐resistant and resist external deformation, whereas at lower internal pressures, the tissue becomes less stiff and ultimately flaccid. When the turgor pressure, and hence, the (compressive) stiffness of the parenchyma tissue decreases, the stiffness of the entire spring‐like tendril also decreases, and, as a consequence, the helical coil relaxes. The tendril‐spring slackens, and the measured tensional forces decrease. Nevertheless, the tendril still continues to coil, so that when the turgor pressure of the parenchyma cells increases after the watering of the plant, the spring is placed under tension and raises the tendril axis minimally but rapidly. This movement can even slightly uncoil the additional turns that had built up in the slack tendril, as observed in our experiments. The reaction to watering occurs rapidly, within several minutes, and indicates efficient water transport from the soil to the tendril. This finding is in agreement with evidence that lianas are highly efficient at being able to conduct water.^[^ ^29^, ^30^, ^31^ ^]^ In addition to the parenchyma, the tendrils contain the lignified fibers that have been discussed above. Although lignification stiffens the cell walls,^[^ ^28^ ^]^ the thin ribbon‐shaped lignified fiber strand found in immature tendrils changes shape slightly when undergoing dehydration showing that the stability of form of the ribbon continues to depend on hydration. The ribbon is therefore not yet fully “frozen” into shape, so that it can still twist and bend when the tendril coils. Indeed, the stabilization of the helical coil in this ontogenetic state does not, or at least not primarily, depend on the lignified ribbon, but on the nonlignified tissues with primary cell walls, as their removal results in a decrease in helix curvature.

Even though these measurements provide strong evidence that the tension in the tendrils during the coiling phase depends directly on the water balance of the plant itself, we can assume that this has no vital influence on the attachment of the stem or of the plant as a whole. In older, fully developed, ontogenetic stages, the tendrils, which are now almost fully lignified, die off and thus become entirely water independent, while still contributing to the overall attachment of the plant stem.

When parenchyma cells of an immature tendril become flaccid because of complete turgor loss, they also coil, indicating that the parenchyma can also act as a resistance layer to the potential G‐fibers in the dehydrated state, via the stiffness of the dried material rather than the structural (i.e., hydrostatic) stiffness in the turgid state. On the other hand, as complete dehydration strongly affects the parenchyma structure, the observed shape change might also be based on a different mechanism from the active coiling in the turgid state.

However, the helical shape resulting from the dehydration‐induced coiling differs from the turgid helix, namely the spring's pitch and radius decrease (compare Figure 2b day 12.5 with days 15 and 17.5), so that the generated tension is lost. In addition to losing intrinsic tension, this might entail a loss of the energy‐dissipating capacity and, thus, of the “advantageous” mechanical function for the plant. Nevertheless, as the dehydration‐induced coiling represents a passive and reversible motion based solely on the hygroscopic properties of the tissue, a better understanding of this mechanism would be valuable not only from a purely botanical viewpoint, but also from a material science perspective in order to explore the tendril as a hydration‐actuated shape‐memory structure.

During ontogeny, lignification proceeds across the entire central cylinder. In contrast to the thin ribbon, the resulting lignified cylinder now fully retains its coiled shape when isolated, i.e., the spring shape in fully developed mature tendrils is structurally “frozen.” Lignin itself is not particularly resistant to tension stress, but the presence of lignin reduces water infiltration and the water content of the cell walls.^[^ ^28^ ^]^ Since dry cellulose is considerably stiffer than wet cellulose, lignification stiffens the cell wall and stabilizes its mechanical properties against fluctuations in water content.^[^ ^28^ ^]^ As a consequence, the tension generated by the coiling tendril becomes increasingly independent of the water status of the plant as lignification proceeds during maturation. Furthermore, with the increasing stiffness of tissues comprising the tendril, we expect that also the stiffness of the tendril‐spring as a whole increases. A stiffer spring can carry heavier loads and provides better energy dissipation, since it deflects less under a given load. This is important as the plant weight increases over time because of leaf growth, the emergence of flowers, and the production of fruits.

In contrast to the hydrostatic, unlignified, thin‐walled parenchyma cells, the thick‐walled lignified cells do not collapse when dehydrated, so that in the mature state, the spring‐shape of the tendril barely changes under water stress. As is known from other passion flower species, the tendrils, in a final step during ontogeny, dehydrate, and completely dry out in the natural process of senescence, while still maintaining a high degree of functionality.^[^ ^32^ ^]^ This can be interpreted as an “energy‐saving mechanism” minimizing the long‐term metabolic energy costs for the plant. Observations of adult plants in the Botanic Garden Freiburg show that in *P. caerulea*, subsequent to the mature state investigated here, the tendrils become senescent while maintaining mechanical functionality. Lignification of the full central cylinder might indeed be the key to enable this dehydration, while avoiding large changes in spring‐shape and, thus, significant losses in mechanical functionality.

**Figure 6:**
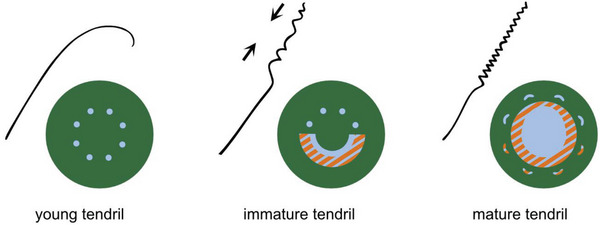
Model for two‐phasic spring formation in *P. caerulea* tendrils. After attachment of the tendril tip, the immature tendril first undergoes an active phase of motion, during which it coils along its axis and thus lashes the plant to its support. In the second phase, the spring‐shape resulting from the coiling is stabilized and further stiffened by lignification of the entire central cylinder. The representations simplify the distribution of lignified (light blue) and parenchymatous (green) tissues, as well as potential G‐fibers (hatched orange).

In brief, the tendril springs in *P. caerulea* form in two phases (**Figure** **6**). First, during an active movement phase after the tendril made contact with a host, it hoists the plant stem toward the host in an upward direction, and forms its spring‐like shape. This phase then transitions into a stabilization phase in which the entire central cylinder lignifies. This two‐step process has inspired the development of artificial tendril and attachment devices (e.g.,^[^ ^33^, ^34^ ^]^).

## 4 Conclusion

We conclude that 1) the coiling tendrils of *P. caerulea* generate forces large enough to move a plant stem and to carry its load, and 2) that the generated tension strongly varies with the water status of the plant and, thus, the turgor pressure of its cells. The main reason for this effect is that the tendril spring is predominantly parenchymatous, and the hydrostatic stiffness of parenchyma depends on its turgor pressure. In this state, 3) the parenchyma stabilizes the coiled shape and the generated tension. In addition, a lignified ribbon of xylem fibers develops unilaterally in the tendril during coiling. We have found first indications that both these xylem fibers and the phloem fibers, which develop later during ontogeny, could be contractile G‐fibers; this framework suggests the unilateral contraction of the bilayer as being the possible motor for tendril coiling. Rather than being persistent, the ribbon‐like shape of the lignified xylem fiber strand merely represents a transitional ontogenetic phase. In addition to active coiling, the tendril in this state also coils significantly when undergoing drying, i.e., in a passive hygroscopic manner. However, it loses its mechanical functionality while drying, as the initial taut spring shape is lost, as is the generated tension and the spring properties.

In a second ontogenetic phase, which can be regarded as a stabilization phase, the lignification progresses beyond the fiber ribbon, so that the shape of the lignified tissue changes from a relatively thin ribbon to a full cylinder. This renders the shape of the spring independent of the existence of a hydrated parenchyma, probably entailing several advantages: i) the generated force becomes more independent of the turgor pressure, ii) the spring stiffens, i.e., it can bear greater loads, and iii) the spring dehydrates with a much smaller shape change, so that it can retain a higher amount of mechanical functionality when becoming senescent. With their ontogenetic multifunctionality and hydration‐actuated shape changes, the coiling tendrils thus provide several intriguing features that should be explored for biomimetic transfer extending beyond the coiling motion itself.

## 5 Experimental Section

### Plant Cultivation

For measurements of the coiling force a potted plant of *Passiflora caerulea* was cultivated in a phytochamber, under controlled temperature and humidity conditions (25.7±0.2 °C, 57±1%; mean and standard deviation for conditions logged in the vicinity of the plant pot during coiling force measurements, data logger EL‐SIE‐6+, Lascar electronics, Whiteparish, UK; accuracy ±0.2 °C, ±1.5%). The plant was grown under constant illumination during the entire experimental time, i.e., without any dark periods (UGR19 Highbay RKU‐200W‐NW LEDs, LeuchTek GmbH, Ahrensburg, Germany), and the light level at the plant was between 120 and 140 µmol m^−2^ s^−1^ PPFD (light meter LI‐250, LI‐COR Biosciences GmbH, Lincoln, USA). Mealybugs were occasionally spotted on the plants but were immediately brushed off using a paintbrush. Shoots for the measurement of their fresh weight were collected from potted *P. caerulea* plants grown in the greenhouse of the Botanic Garden of the University of Freiburg, and flowers from the potted plant cultivated in the phytochamber. For histology and dehydration‐rehydration experiments, tendrils were collected from *P. caerulea* plants cultivated in either the greenhouses or outdoor areas of the Botanic Garden.

### Coiling Force Measurement

**Figure 7:**
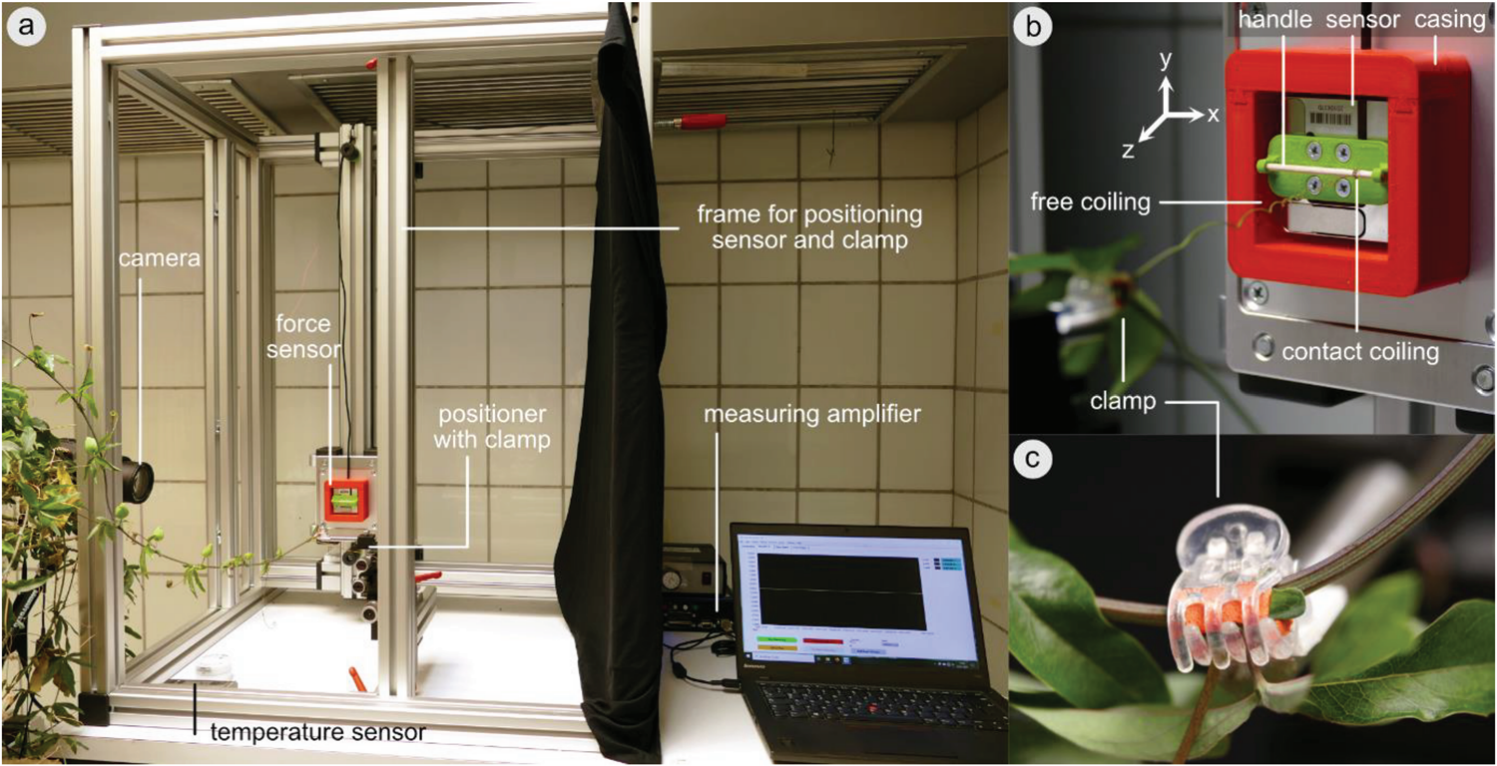
Experimental setup. To measure the force generated by the free coiling of *P. caerulea* tendrils, a custom‐built measuring setup consisting of a 3‐axes‐force sensor was used a), which was equipped with a handle, around which the tendril apex coiled (contact coiling) b), and a positioner with a clamp, which retained the stem at the tendril base c).

The coiling force of four *P. caerulea* tendrils was measured using a custom‐build setup (**Figure** **7**). Whereas three measurements served primarily for studies of the maximum force produced, one tendril was analyzed with the aim of additionally investigating the influence of the plant's hydration status on the coiling force. During this measurement, soil humidity was tracked continuously during tendril coiling, and the coiled tendril was cut off from the plant and left to dry within the setup.

The setup consisted of a 3‐axis‐force sensor (K3D40 ±2N, accuracy class 0.5%), in combination with a measuring amplifier (GSV‐8DS SubD44HD, both ME‐Meßsysteme GmbH, Henningsdorf, Germany) and the manufacturer's software (GSVmulti version 1.46.1 2020) (Figure 7). The sensor was attached to a movable mount within a sturdy aluminum frame, allowing the free positioning of the sensor within a 2D‐plane. On the opposite side of the frame, a micropositioner was positioned equipped with a 1 cm hairgrip clamp padded with a piece of craft foam sheet, to immobilize the shoot and thus to prevent it from tensioning the tendril by its own weight. A custom‐made 3D‐printed holder (ABS, Acrylonitrile butadiene styrene) with a small handle consisting of a bamboo toothpick (Wenco‐Service Marketing GmbH & Co. KG, Essen, Germany) was mounted on the live end of the sensor by using four cylindrical head screws. The handle was 2 mm in diameter and 37 mm in length, and the distance between the handle's central axis and the back of the holder, i.e., the sensor surface, was 6.5 mm. An uncoiled tendril with a slightly bent tip was carefully and loosely placed on the handle. The stem was clamped at the tendril's base. Both the apex and the leaf at the clamped node developed normally, i.e., clamping did not disturb the development of the plant. The tendril was continuously photographed at a frequency of 1 image per minute (Lumix DMC‐FZ1000, Panasonic, Kadoma, Japan), and the individual images were combined to produce a time‐lapse video. Temperature, relative humidity, and air pressure were logged at 1/60 Hz (data logger EL‐SIE‐6+, Lascar electronics, Whiteparish, UK; accuracy ±0.2 °C, ±1.5%, ±1 mbar). All potential disturbances of the measurement (by people entering the chamber to attend to the experiments or to water the plant pots) were carefully noted. The force along each of the *x*‐, *y*‐, and *z*‐axis was measured with a sampling frequency of 1 Hz, which was reduced to 1/60 Hz for subsequent analysis of the data. From the three force channels, the total force parallel to the coiled tendril's length axis was calculated as $F\;=\sqrt{Fx^{2}+Fy^{2}+Fz^{2}}$.

For the measurement targeting the hydration effects on the coiling force, the soil humidity in the plant pot was continuously tracked (one measurement every 15 min; sensor TMS‐4, TOMST s.r.o., Prague, Czech Republic;^[^ ^35^ ^]^ the data regarding the raw moisture count are reported). To quantify the degree of coiling, the rotational motion of the perversion visible in the time‐lapse video (Video S2, Supporting Information) was analyzed, and the number of turns that it performed was counted in 0.25 steps. Any reversal in the turning direction of the perversion was documented (uncoiling), and the turns were counted, rounded to 0.25 turns. After 14.2 days, the plant stem was cut apically and basally to the clamped section leaving the coiled and still clamped tendril to dry out in its original position and thus allowing the force measurement to be continued. With regard to the drying tendril, the degree of coiling was quantified as described above, except that the number of turns that the perversion performed was counted in larger steps. After the tendril had been left to dry in the setup for 4.8 days, it was removed from the setup for further analyses in the laboratory. A small segment from the coiled part of the dried tendril was rehydrated in tap water for 30 min and photographed (Lumix camera). From the remaining dried tendril, three segments from the coiled part and one segment from the straight basal part were embedded and thin sectioned, as described in the following section.

### Histology

Based on preliminary experiments, it was distinguished between young, immature and mature tendrils depending on the development of their anatomical features (cf. results section). From each developmental stage, tendrils were collected for i) lignin‐specific phloroglucinol‐hydrochloric acid staining of fresh sections,^[^ ^36^ ^]^ ii) for embedding in resin, allowing thin‐sectioning and a precise alignment of the coiled part of the tendril with respect to its length axis, and iii) dual staining of cyrotome sections using astrablue‐safranin. Moreover, a mature tendril was collected for the immunolocalization of RG‐I (rhamnogalacturonan‐I) by using the primary antibody LM6 ([Anti‐1,5‐α‐L‐Arabinan] Antibody, Megazyme Ltd, Bray, Ireland) and a fluorescent marker (Alexa Fluor 568 goat antirat IgG (H+L), Thermo Fisher Scientific Inc., Waltham, MA). The supplement provides the full protocol. All immature and mature tendrils were sectioned within the coiled tendril part.

For lignin detection, fresh sections were cut with a razor blade and were subsequently submerged briefly in a phloroglucinol‐ethanol solution (wt 1%) and then briefly in 20% hydrochloric acid. For microscopy, sections were transferred to tap water on a slide and photographed (microscope Primostar with camera Axiocam eRc 5s, both Zeiss, Oberkochen, Germany).

For thin sectioning of embedded samples, tendril material was fixed in FAA, namely a mixture of 70% ethanol, 37% formaldehyde solution, and glacial acetic acid (at a ratio of 90:5:5) for 2–48 days. Samples were then dehydrated through a series of increasing isopropanol concentrations and embedded in resin (Technovit 7100, Kulzer GmbH, Hanau, Germany). Sections were obtained using a rotary microtome (CUT 5062, SLEE medical GmbH, Nieder‐Olm, Germany), stained with toluidine blue (0.05% (wt)), and transferred to glass slides. For microscopy, the slides were sealed with Entellan (Merck, Darmstadt, Germany), and the sections were photographed (microscope BX61 with camera DP71, both Olympus, Tokio, Japan).

For dual staining with astrablue‐safranin stain, fresh tendrils were embedded in Tissue‐Tek O.C.T. Compound (Sakura Finetek, Tokyo, Japan) and sectioned at 40 µm thickness on a cryostat (SLEE Cryostat MEV, SLEE medical GmbH, Mainz, Germany). Sections were bleached (10% Eau de Javel, ≈10 min) and washed (2x a minimum of 1 min in deionized water), and stained as follows: safranin (1 g safranin O in 100 mL deionized water) 9–12 s, a wash in deionized water until no more color washed out, astrablue (2 g tartaric acid and 0.5 g astrablue in 100 mL deionized water) 11–15 s, and finally, a wash in deionized water until no more color washed out. Sections were then transferred to fresh deionized water and photographed (microscope BX61 with camera DP71).

### Dehydration‐Rehydration Experiments

To investigate the effect of the hydration status on the shape of the coiled tendril and on isolated lignified structures, two mature and immature tendrils were collected. A coiled segment was cut from the distal end of each tendril. Lignified tissues were isolated from one tendril per ontogenetic state. To this end, the segment was incubated with the enzyme Driselase for 72 h at 22–37 °C (based on^[^ ^18^ ^]^) (Sigma‐Aldrich, MO; 2% (wt) Driselase solved in phosphate‐buffered saline). Samples were then sonicated for 20 min (ultrasonic cleaner Emmi H60, EMAG AG, Mörfelden‐Walldorf, Germany), and any remaining parenchyma tissue material stuck to the lignified structures was gently removed using a brush. The isolated lignified structure was photographed (cf. Figure 5b1,d1) (stereomicroscope SZX9, Olympus, Tokio, Japan with camera Color View II, Soft Imaging System GmbH, Münster, Germany). Both the treated tendril segments and the fresh segments from each developmental stage were fixed at one end, air‐dried for 48 h, and subsequently submerged in tap water for another 48 h. The drying and rehydration process was photographed (1 picture min^−1^, Lumix DMC‐FZ1000, Panasonic, Kadoma, Japan).

### Measurement of Fresh Weight of Plant Material

To determine the fresh weight of the plant material, 37 plant stem segments of 1 m were harvested, including the leaves. The number of nodes in each segment was counted, and the segment was weighed, enabling the mean weight per node to be determined. Additionally, nine flowers with stalks were cut and weighed.

## Conflict of Interest

The authors declare no conflict of interest.

## Supporting information

Supporting Information

Supplemental Video 1

Supplemental Video 2

Supplemental Video 3

## Data Availability

All data supporting the findings of this study are available within the paper and its supplementary information files, or are available from the corresponding author (Frederike Klimm) upon reasonable request.

## References

[1] C. Darwin , The Movements and Habits of Climbing Plants, John Murray, London, 1882.

[2] K. C. Vaughn , A. J. Bowling , in Horticultural Reviews (Ed: J. Janick ), John Wiley & Sons, Inc., Hoboken, NJ 2011, p. 1. 10.1002/9780470872376.ch1.

[3] N. P. Rowe , T. Speck , in Ecology of Lianas (Eds: S. A. Schnitzer , F. Bongers , R. J. Burnham , F. E. Putz ), John Wiley & Sons, Ltd, Chichester 2015, pp. 323–341. 10.1002/9781118392409.ch23.

[4] T. Steinbrecher , G. Beuchle , B. Melzer , T. Speck , O. Kraft , R. Schwaiger , Int. J. Plant Sci. 2011, 172, 1120.

[5] H. F. Bohn , F. Günther , S. Fink , T. Speck , Int. J. Plant Sci. 2015, 176, 294.

[6] M. J. Jaffe , A. W. Galston , Annu. Rev. Plant Physiol. 1968, 19, 417. 10.1104/pp.43.4.537 PMC1086884 16656803

[7] D. T. MacDougal , Bot. Gaz. 1893, 18, 123.

[8] A. Goriely , M. Tabor , Phys. Rev. Lett. 1998, 80, 1564.

[9] T. McMillen , A. Goriely , J. Nonlinear Sci. 2002, 12, 241.

[10] D. T. MacDougal , Ann. Bot. 1896, 10, 373.

[11] M. J. Jaffe , A. W. Galston , Plant Physiol. 1966, 41, 1014. 16656344 10.1104/pp.41.6.1014 PMC1086466

[12] A. J. Bowling , K. C. Vaughn , Am. J. Bot. 2009, 96, 719. 21628227 10.3732/ajb.0800373

[13] T. Gorshkova , Carbohydr. Polym. 2022, 295, 119849. 35988975 10.1016/j.carbpol.2022.119849

[14] T. Alméras , B. Clair , J. R. Soc. Interface 2016, 13, 20160550. 27605169 10.1098/rsif.2016.0550 PMC5046956

[15] B. Clair , T. Alméras , G. Pilate , D. Jullien , J. Sugiyama , C. Riekel , Plant Physiol. 2011, 155, 562. 21068364 10.1104/pp.110.167270 PMC3075793

[16] N. Schreiber , N. Gierlinger , N. Pütz , P. Fratzl , C. Neinhuis , I. Burgert , Plant J. 2010, 61, 854. 20030750 10.1111/j.1365-313X.2009.04115.x

[17] C. G. Meloche , J. P. Knox , K. C. Vaughn , Planta 2007, 225, 485. 16955273 10.1007/s00425-006-0363-4

[18] S. J. Gerbode , J. R. Puzey , A. G. McCormick , L. Mahadevan , Science 2012, 337, 1087. 22936777 10.1126/science.1223304

[19] J. G. Chery , R. A. E. Glos , C. T. Anderson , New Phytol. 2022, 233, 126. 34160082 10.1111/nph.17576

[20] J.‐S. Wang , G. Wang , X.‐Q. Feng , T. Kitamura , Y.‐L. Kang , S.‐W. Yu , Q.‐H. Qin , Sci. Rep. 2013, 3, 3102. 24173107 10.1038/srep03102 PMC3813933

[21] A. Eberle , K. Quinn , L. Bassman , in Proceedings of the Sixth Plant Biomechanics Conference (Ed: B. Thibaut ), Cayenne, French Guyana, 2009, pp. 67–74.

[22] N.‐S. Liou , G.‐W. Ruan , H.‐H. Yen , Strain 2013, 49, 16.

[23] T. Ulmer , J. M. MacDougal , Passiflora. Passionflowers of the World, Timber Press, Inc, Portland, OR 2004.

[24] C. Feuillet , J. M. MacDougal , in The Families and Genera of Vascular Plants (Ed: K. Kubitzki ), Springer, Heidelberg 2007, pp. 270–281. 10.1007/978-3-540-32219-1_35.

[25] D. T. MacDougal , Bot. Gaz. 1892, 17, 205.

[26] A. J. Bowling , K. C. Vaughn , Am. J. Bot. 2008, 95, 655. 21632390 10.3732/ajb.2007368

[27] O. A. Patova , V. V. Golovchenko , Y. S. Ovodov , Russ. Chem. Bull. 2014, 63, 1901.

[28] K. J. Niklas , Plant Biomechanics. An Engineering Approach to Plant Form and Function, The University of Chicago Press, Chicago 1992.

[29] F. W. Ewers , IAWA J. 1985, 6, 309.

[30] F. W. Ewers , J. B. Fisher , Oecologia 1991, 88, 233. 28312137 10.1007/BF00320816

[31] B. L. Gartner , Oecologia 1991, 87, 180. 28313834 10.1007/BF00325255

[32] F. Klimm , S. Schmier , H. F. Bohn , S. Kleiser , M. Thielen , T. Speck , J. Exp. Bot. 2022, 73, 1190. 34673926 10.1093/jxb/erab456 PMC8866636

[33] F. Meder , S. P. Murali Babu , B. Mazzolai , IEEE Robot. Autom. Lett. 2022, 7, 5191.

[34] T. Speck , T. Cheng , F. Klimm , A. Menges , S. Poppinga , O. Speck , Y. Tahouni , F. Tauber , M. Thielen , MRS Bull. 2023, 48 , 1.

[35] J. Wild , M. Kopecký , M. Macek , M. Šanda , J. Jankovec , T. Haase , Agric. Meteorol. 2019, 268, 40.

[36] D. Gerlach , Botanische Mikrotechnik, 3rd ed., Georg Thieme, Stuttgart 1984.

